# 34 Evaluation Experiences from The Healthy & Active Fund​ in Wales

**DOI:** 10.1093/eurpub/ckae114.166

**Published:** 2024-09-26

**Authors:** Vasiliki Kolovou, Anna Kolosowska, Rochelle Embling, Niamh Mchugh, John Bradley, Paul Pilkington

**Affiliations:** Health Improvement Division, Public Health Wales, United Kingdom; Health Improvement Division, Public Health Wales, United Kingdom; Health Improvement Division, Public Health Wales, United Kingdom; Health Improvement Division, Public Health Wales, United Kingdom; Health Improvement Division, Public Health Wales, United Kingdom; Health Improvement Division, Public Health Wales, United Kingdom

## Abstract

**Purpose:**

To explore diverse perspectives of stakeholders involved in the evaluation of community-based projects. Third-sector organisations and public sector bodies based across different regions in Wales, delivered a variety of physical activity and mental well-being interventions, as part of the Healthy and Active Fund programme. Projects designed and completed evaluations of their process and outcomes, and their experiences offer lessons to inform future community-based delivery to under-served populations.

**Methods:**

Semi-structured interviews (N = 14) were completed with project members (N = 15, 12 projects) that had roles in leading, managing or completing aspects of the project-level evaluations. Interviews were analysed according to reflexive thematic analysis and themes were collaboratively developed.

**Results:**

Three major themes were identified across interviews that were relevant to the lessons learnt from the evaluation of community-level projects and delivering to under-served populations: structural level of support, capturing impact, and adapting and learning. All projects were required to use questionnaire-based, self-reported physical activity and mental well-being outcome measures (IPAQ and WEMWBS), while some projects collected additional data through interviews, focus groups and questionnaires. Projects acknowledged the importance of robust evaluation of their activities, but for many organisations and members it was the first time they had planned evaluations of that scope and level of detail. The support provided from the Healthy and Active Fund programme was valuable. Some projects needed closer guidance and problem-solving, despite most projects having sought external evaluation expertise to plan or conduct evaluations of their projects.

**Conclusions:**

This study revealed several barriers and facilitators for engaging under-served populations in monitoring and evaluation, especially in using standard questionnaires. The lessons from these projects can inform future community-based approaches to evaluation and encourage a culture of evaluation and monitoring as standard practice. Such evaluation and monitoring can assist third-sector organisations and public bodies with showcasing their impact and securing ongoing funding.

**Support/Funding Source:**

None. Conducted by Public Health Wales.

